# Non-Invasive Assessment of Tertiary Lymphoid Structure in Hepatocellular Carcinoma Based on Multi-Parameter Imaging: From Tumor Immunobiology to Immunotherapy Benefit Prediction

**DOI:** 10.3390/cancers18182978

**Published:** 2026-09-15

**Authors:** Dan Liu, Qian Li, Feng Che, Qin Wang, Tong Zhang, Wei Ren, Liping Deng, Bin Song, Yi Wei, Hehan Tang, Jing Zhu, Yuan Yuan

**Affiliations:** 1 Department of Radiology, West China Hospital, Sichuan University, Chengdu 610041, China; 2 CT Imaging Research Center, GE Healthcare China, Beijing 100176, China

**Keywords:** tertiary lymphoid structure (TLS), hepatocellular carcinoma (HCC), multi-parametric imaging, immunotherapy, prognosis prediction

## Abstract

Tertiary lymphoid structures (TLSs) are ectopic lymphoid aggregates within the intratumoral and peritumoral microenvironment. In hepatocellular carcinoma (HCC), the presence of TLSs was closely associated with immunotherapy response and prognostic outcomes. However, TLSs exhibit pronounced spatial heterogeneity within tumors and biopsy, being invasive and prone to sampling bias, may cause inaccurate assessments. Multi-parametric imaging techniques based on computed tomography (CT)/magnetic resonance imaging (MRI)CT/MRICT/MRI with the aid of artificial intelligence (AI) algorithms could effectively integrate anatomical structures with functional metabolic information, quantifying and visualizing key pathological molecular features of tumor treatment response across multiple dimensions. This review summarizes the biological characteristics and clinical significance of TLSs in hepatocellular carcinoma. Furthermore, it focuses on the latest advances in non-invasive TLS assessment using multi-parametric imaging techniques, integrating these technologies with large-scale artificial intelligence models to explore their application value in prognosis prediction and immunotherapy benefit evaluation.

## Simple Summary

Hepatocellular carcinoma is a deadly liver cancer where immunotherapy helps only some patients. Tertiary lymphoid structures are immune cell clusters in and around tumors that predict immunotherapy response, but detecting them currently requires invasive biopsies that risk missing these structures due to tumor heterogeneity. This review summarizes the biological characteristics and clinical significance of TLSs in hepatocellular carcinoma, with emphasis on recent advances in non-invasive TLS assessment via multi-parametric imaging. It further explores the integration of these imaging technologies with large-scale AI models for prognostic prediction and immunotherapy response evaluation.

## 1. Introduction

Hepatocellular carcinoma (HCC) is the predominant histological subtype of primary liver cancer. In China, it ranks fourth among malignant tumors in incidence and second in mortality. Annually, it accounts for over 50% of new cases and deaths worldwide, imposing a significant burden on public health and the economy [1]. Over 70% of HCC patients in China are diagnosed at an intermediate or advanced stage, losing opportunities for curative treatment and facing poor prognosis. The introduction of immune checkpoint inhibitors (ICIs) has significantly transformed the treatment landscape for hepatocellular carcinoma (HCC), with ICIs now established as the standard first-line therapy for advanced HCC [2]. However, because the liver is a highly immunotolerant organ that readily induces immune tolerance rather than immune activation, response rates to immunotherapy vary significantly among individuals. Therefore, identifying HCC patients who are sensitive to immunotherapy and guiding personalized, precision treatment remains an urgent challenge that requires resolution [3,4].

Tertiary lymphoid structures (TLSs) are immune cell aggregates that develop postnatally in non-lymphoid tissues within chronic inflammatory or tumor microenvironments (TMEs), structurally resembling secondary lymphoid organs (SLOs). They are typically absent under physiological conditions but emerge during persistent inflammatory states such as cancer, chronic infections, and autoimmune diseases. HCC patients frequently have a history of chronic viral hepatitis and non-alcoholic steatohepatitis, making TLS structures a distinctive pathological feature within HCC tissue [5]. The presence of tertiary lymphoid structures provides a critical microenvironment for the generation of antitumor immune responses and correlates with improved clinical outcomes in most cancers. In HCC, the presence of tumor-associated tertiary lymphoid structures is associated with a lower risk of early recurrence after surgical resection, suggesting TLSs are promising immunotherapy biomarkers [6,7,8].

Current conventional methods for detecting TLSs include hematoxylin and eosin (H&E) staining, immunofluorescence (IF), multiplex immunohistochemistry (mIHC), and spatial transcriptomics [9]. However, it faces the following limitations: HCC (especially advanced HCC) often relies on non-invasive clinical or imaging diagnosis without requiring pathological confirmation, which partially restricts tumor tissue acquisition and the widespread application of histopathological examination. Furthermore, due to the high heterogeneity of advanced HCC, histopathological examination often relies on localized sampling, making it difficult to comprehensively reflect the entire tumor profile. Different tumor regions may exhibit distinct biological characteristics, and sampling errors introduce limitations to the precise assessment based on tissue samples [10]. Therefore, developing a non-invasive, preoperative TLS prediction tool holds significant clinical importance for individualized treatment decisions. Multi-parametric imaging techniques based on CT/MRI effectively integrate anatomical structures with functional metabolic information, quantifying and visualizing key pathological molecular features of tumor treatment response across multiple dimensions—including morphology, structure, function, and metabolism. This represents an accurate and reliable non-invasive method for assessing the tertiary lymphoid structure of hepatocellular carcinoma. Artificial intelligence (AI) algorithms can delve deeper into structural patterns and specific signals, precisely identifying spatial heterogeneity within lesion areas. Consequently, AI-enhanced multi-parametric imaging has the potential to provide crucial insights into HCC TLS architecture, aiding in precision treatment decision-making and ultimately improving patient prognosis.

This review aims to systematically summarize the biological characteristics and clinical significance of tertiary lymphoid structures (TLSs) in hepatocellular carcinoma. Furthermore, it focuses on the latest advances in non-invasive TLS assessment using multi-parametric imaging techniques, integrating these technologies with large-scale artificial intelligence models to explore their application value in prognosis prediction and immunotherapy benefit evaluation.

## 2. Biological Basis of Tertiary Lymphoid Structures in Hepatocellular Carcinoma

### 2.1. TLS Formation in the HCC Microenvironment

TLSs are not random lymphocytic accumulations [11]; instead, they are ectopic immune organs formed by the coordinated action of chemokine axes, stromal organizers, vascular entry routes and persistent antigenic stimulation [12,13,14,15,16,17]. In HCC, this process tends to be within a continuous liver-tumor immunobiological system rather than as a strictly intratumoral event [9]. Persistent viral antigen exposure, tumor antigen release and damage-associated molecular patterns (DAMPs) arising from chronic liver injury may create a permissive inflammatory context for TLS neogenesis [18,19,20,21,22]. Consistent with this concept, a CT-based study found that positive HBsAg or HCV antibody status was independently associated with iTLS presence (OR = 4.52, 95% CI: 1.65–13.29; *p* = 0.004) in HCC, supporting a link between chronic liver disease background and TLS-related immune organization [23]. This association may suggest that liver disease etiology and tumor immune architecture may be biologically associated.

#### 2.1.1. Stromal and Vascular Organization

Following the establishment of a chronic inflammatory environment, stromal and vascular remodeling provides an important structural basis for TLS organization. The lymphotoxin-α1β2–lymphotoxin-β receptor (LTα1β2–LTβR) axis promotes the differentiation of local stromal cells, including fibroblasts and perivascular mural cells, toward an organizer-like phenotype capable of maintaining homeostatic chemokine programs. In parallel, inflammatory TNF/TNFR cues frequently cooperate to induce adhesion molecules such as VCAM-1 and ICAM-1. This enables stable leukocyte recruitment and compartmentalization [24,25,26].

A key liver-specific feature is the prevalence of fibrosis and hepatic stellate cell (HSC) activation, which may amplify responsiveness to LTβR signaling. In a chronic liver injury model, Ruddell et al. [27] proposed that LTβR signaling in HSCs contributes to paracrine recruitment and progenitor/leukocyte mobilization, providing a mechanistic basis for the coupling between fibrotic remodeling and immune organization. This framework may help explain why TLSs are frequently observed near fibrous septa and portal tracts, where stromal scaffolds and vascular structures may provide a permissive environment for lymphoid organization.

These stromal and vascular alterations may also contribute indirectly to imaging-visible characteristics of HCC. For example, fibrous capsule or pseudocapsule formation can produce characteristic rim or capsule enhancement patterns on CT and MRI [28,29]. Beyond such capsules, peritumoral enhancement and hepatobiliary-phase signal patterns may reflect broader invasive-margin biology rather than lymphoid organization alone. For example, rim arterial-phase hyperenhancement, arterial-phase peritumoral enhancement and intermediate hepatobiliary-phase intensity were associated with immune-excluded HCC, with OR of 17.3, 8.6 and 28.2, respectively. Spatial transcriptomics further localized CAF-, VEGF- and CD8-related signals around the tumor-invasive margin [30]. However, although these imaging features may reflect stromal remodeling and the tumor–liver interface, they should not be interpreted as direct imaging markers of TLSs. Current evidence supports a biologically plausible association between stromal organization and imaging phenotype, but does not establish a direct pathway-level correspondence between LTβR-, TNF/TNFR-, or chemokine-driven stromal programs and specific CT/MRI findings. Therefore, this relationship requires further validation using spatially matched radiology–pathology and radiology–spatial-omics approaches [28,29,30,31].

#### 2.1.2. Chemokine-Mediated Lymphocyte Recruitment and Spatial Organization

Once a stromal and vascular framework has been established, chemokine signaling contributes to the recruitment and spatial organization of lymphocytes within TLSs. The CXCL13–CXCR5 axis plays an important role in recruiting B cells and organizing B-cell follicles, whereas the CCL19/CCL21–CCR7 axis promotes the recruitment of T cells and CCR7-positive migratory or mature dendritic cells (DCs) and contributes to their localization within T-cell compartments [32,33]. The coordinated activity of these chemokine pathways is essential for transforming a nonspecific lymphoid aggregate into an organized immune structure. Importantly, a mature TLS is characterized not only by the presence of B cells, but also by the emergence of an integrated architecture. This includes follicular dendritic cell (FDC) networks, germinal center (GC) reactions and high endothelial venules (HEVs), which enable local antigen presentation, clonal expansion and coordinated humoral-cellular immunity [11,13].

### 2.2. TLS Maturation and Functional Organization

TLSs are dynamic structures that undergo progressive maturation rather than representing a single uniform histological entity. Their functional activity depends not only on their presence but also on the degree of cellular organization, follicular development, and vascular specialization [34,35,36,37,38].

#### 2.2.1. The TLS Maturation Continuum

A synthesis of TLS maturation frameworks has emphasized that coupling between LT signaling and chemokine circuitry is crucial for compartment formation and functional maturation [35]. TLS research has focused on formation, whereas the immunotherapy era demands understanding TLS trajectories. Shu et al. [36] identified a non-canonical involuted TLS morphology in HCC treated with neoadjuvant immunotherapy, characterized by B-follicle disaggregation while preserving T-zone and DC-T interactions, alongside upregulation of memory-associated signatures. This observation reframes TLS from a static histologic structure to a dynamic immune process that can be measured. It also provides a decisive biological anchor for non-invasive evaluation, suggesting that imaging may need to detect not merely TLS presence, but TLS state along the life-cycle continuum. TLSs exhibit a continuum of maturation states that are commonly categorized into three stages [38]. Immature TLSs are dense but poorly organized lymphocytic clusters lacking FDC networks and GC reactions. Primary follicle-like TLSs display an FDC network, whereas secondary follicle-like TLSs represent mature TLSs with active GC features and are typically associated with PNAd+ HEVs, which is consistent with enhanced lymphocyte trafficking [11]. Clinically, TLS maturity tends to be more closely associated with functional antitumor immunity than the presence of TLSs alone [12,34,37]. Consequently, the maturation continuum of TLSs may create an important reference-standard problem for imaging studies. A binary pathological label, such as TLS-positive/TLS-negative, likely compresses several biologically non-equivalent states, including early lymphoid aggregates lacking FDC/GC organization, GC-competent TLS with HEV-associated lymphocyte trafficking, and therapy-remodeled or involuted TLS after immune checkpoint blockade. This concern is supported by recent spatial and treatment-response studies showing that TLS maturation is not linear: spatial transcriptomics has suggested that tryptophan-enriched tumor metabolism can restrict iTLS maturation, whereas neoadjuvant immunotherapy may reshape TLSs into divergent morphologies with preserved T-zone/DC interactions but disaggregated B-cell follicles [36,39]. Therefore, imaging-based TLS assessment should be framed as an attempt to approximate TLS-related immune-stromal states rather than merely TLS presence. However, because routine imaging cannot directly resolve FDC networks, germinal centers or PNAd+ HEVs, the current evidence supports this as a methodological direction rather than proof that conventional imaging can already discriminate TLS maturity directly.

#### 2.2.2. Cellular and Vascular Components of Mature TLSs

TLSs comprise distinct immune cell modules that partially recreate the microanatomy of secondary lymphoid organs [11,13]. (1) B-cell follicles/GCs: mature TLSs may contain CD20+ follicles, and GC competence is supported when follicular organization occurs alongside an FDC network and GC markers, consistent with local affinity maturation and humoral activity [40,41]. (2) T-cell zones: interspersed T-cell areas contain CD4+ and CD8+ subsets and are typically organized by the CCL19/CCL21-CCR7 axis that co-localizes T cells with CCR7+ migratory/mature DCs. In contrast, CXCL13 predominantly structures adjacent B follicles and recruits CXCR5+ Tfh [42,43]. (3) Antigen-presenting cells: mature DCs occupy the T-cell compartment and/or the T-B interface to sustain local antigen presentation and adaptive immunity, complementing draining lymph node priming [13,44]. (4) HEVs: PNAd (MECA-79)+ HEVs are often found in mature TLS and enable the selective entry of lymphocytes, supporting the sustained recruitment and retention of effector cells [45,46]. Among these components, HEVs provide the most biologically plausible bridge to perfusion-related imaging. Furthermore, they provide the most plausible biological link to perfusion-related imaging, but the relationship should be framed cautiously. PNAd/MECA-79+ HEVs are often found in mature TLSs and enable selective lymphocyte entry. In resected HCC, tumor-associated HEVs were identified in 31/156 cases, increased with TLS maturation, and were associated with higher intratumoral CD4+/CD8+ T-cell densities and better 3-year disease-free survival (79.8% vs. 60.1%, *p* = 0.011) [47]. However, HEVs are specialized post-capillary venules rather than tumor-feeding arteries, and current CT/MRI cannot directly depict them. Consistently, CT and MRI studies suggest that iTLS-positive or mature iTLS tumors tend to show fewer intratumoral arteries and fewer hemorrhagic or aggressive vascular features, rather than simply stronger arterial enhancement [23,48]. Thus, HEV-containing TLSs may be more reasonably linked to an organized immune-permissive vascular niche than to a single conventional enhancement sign.

### 2.3. Heterogeneous Subgroups of TLSs

Both the relative abundance and maturation state of TLS constituents affects their functionality [35,38]. Immature lymphoid aggregates lacking HEVs and GC/FDC organization probably represent early lymphoid neogenesis, characterized by reduced capacity for sustained lymphocyte recruitment and limited GC-dependent humoral maturation [34,35]. In contrast, mature TLSs which are characterized by organized follicles, GC features and HEV-rich vasculature are more consistently associated with active local antigen presentation and coordinated amplification of humoral-cellular immunity, complementing priming in draining lymph nodes [11,37,45].

According to the spatial localization, TLSs are typically classified by spatial location into intratumoral TLSs (iTLSs) and peritumoral TLSs (pTLSs). iTLSs are located within the tumor parenchyma, while pTLSs are generally identified at the tumor–liver interface, including the tumor periphery, invasive margin or adjacent non-tumor tissue. pTLSs may better reflect the background immune reaction at the liver–tumor interface, whereas iTLSs may indicate whether the immune system has successfully penetrated the tumor core. In this review, pTLS is used as a topographic descriptor for a TLS located around the tumor–liver interface, rather than as a fixed-distance pathological category. Therefore, when individual studies are compared, the original sampling boundary should be explicitly reported. For example, Li et al. quantified pTLS density within 5 mm from the infiltrative tumor border and reported that iTLSs were present in 31.7% of tumors, whereas pTLSs were nearly ubiquitous (95%) [49]. In contrast, Zhang et al. analyzed TLSs in peritumoral tissue sampled approximately 1 cm from the tumor edge and reported that higher pTLS burden was associated with inferior prognosis and increased neutrophil infiltration [50]. In addition, Sarelin et al. quantified pTLSs in HCC using three methods: pTLS density, defined as the number of pTLSs within 3 mm of the invasive front divided by the length of the invasive front; maximum pTLS diameter; and pTLS hotspot count, defined as the number of pTLSs within a 5-mm hotspot area [51]. This discrepancy may partly reflect distance-dependent sampling of different compartments: a 5 mm invasive-border region may be anatomically closer to the tumor–liver interface, whereas a 1 cm region may include a broader adjacent-liver compartment influenced by chronic inflammation, fibrosis and field effects. This interpretation remains inferential, but it is supported by a recent HCC meta-analysis showing that pTLS-related prognostic results were unstable and that differences in peritumoral tissue extent, including 1 cm versus 5 mm sampling, were an important source of heterogeneity [51]. It is also biologically plausible because HCC-adjacent tissues commonly exhibit cirrhosis or fibrosis, and proteomic studies indicate that the peritumor microenvironment differs substantially from the intratumoral microenvironment [52]. Consequently, future pathology and radiomics studies should specify whether the analyzed region corresponds to the invasive front, a 5 mm rim, a 1 cm tumor-free region or another predefined peritumoral compartment. This requirement is consistent with the broader recommendation that peritumoral tissue studies should standardize naming and document sampling distance from the tumor border [53].

### 2.4. Context-Dependent Clinical Significance of TLSs

**Figure 1:**
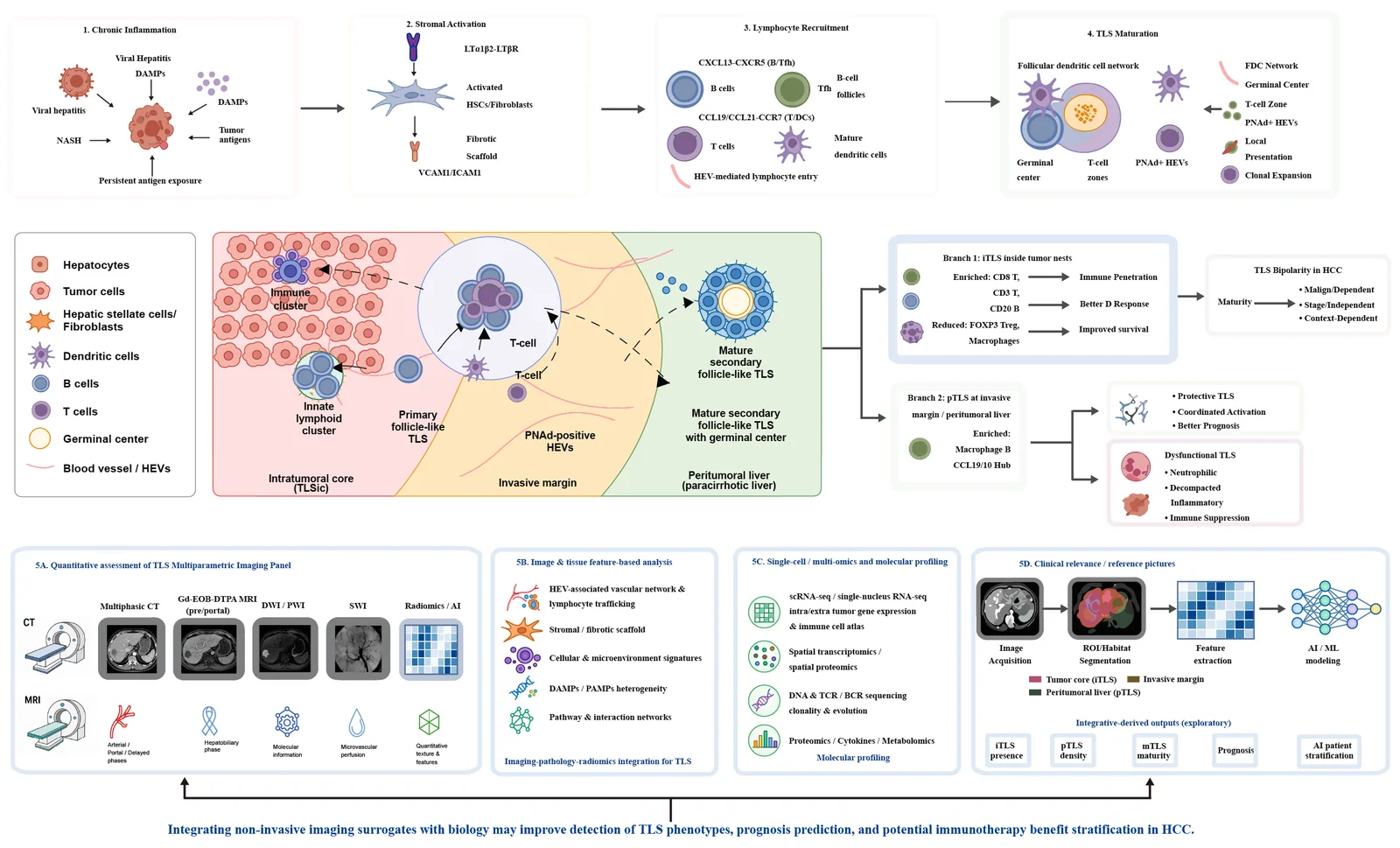
Biological basis and imaging-derived surrogate assessment of TLSs in hepatocellular carcinoma.

In HCC, TLSs are frequently linked to improved clinical outcomes, consistent with a microenvironment permissive for effective antigen presentation and T-cell activation [8,54,55]. However, HCC exhibits unusually strong TLS functional bipolarity: the liver is simultaneously an immunologically active organ and a tissue that is susceptible to tolerance due to chronic antigen exposure [56]. Therefore, TLSs can amplify antitumor immunity when organized toward GC-supported adaptive responses. However, in specific inflammatory conditions, they may align with immunoregulatory programs, thereby attenuating effector responses and facilitating immune escape [20,57]. Accordingly, TLS in HCC should be viewed as a dynamic, context-dependent immune program whose impact can change as the disease progresses. This impact is modulated by TLS maturation, immune composition, spatial localization, underlying liver etiology/inflammatory background and treatment-induced remodeling [11,58], Figure 1 shows the dynamic role of TLSs in HCC.

#### 2.4.1. iTLS and Prognosis

Recent evidence reveals that iTLS and pTLS have different associations with tumor progression, recurrence and immunotherapy response. TLSs more prominently reflected antigen presentation efficiency and cytotoxic T lymphocyte (CTL) activation status within tumor parenchyma [59]. Meanwhile, an iTLS localized within tumor depth indicates effective lymphocyte infiltration and immunoediting processes in local regions [59,60]. iTLS is generally associated with favorable prognosis in HCC patients [8,54]. In a large resection cohort (*n* = 273), iTLS density was correlated with lower early recurrence risk (<2 years) with HR = 0.46 (*p* = 0.005), recurrence risk varied with iTLS maturation, and aggregates were associated with higher early recurrence risk (primary/secondary follicles vs. aggregates; *p* = 0.01) [8]. A meta-analysis [55] further supports the concept: iTLS was associated with longer RFS (HR = 0.60, 95% CI 0.50–0.67; *p* < 0.001) and reduced early recurrence (HR = 0.49, 95% CI 0.36–0.65; *p* < 0.001), while no significant associations were found with OS or late recurrence in pooled estimates. Mechanistically, iTLS positivity was accompanied by increased intratumoral CD3+, CD8+, CD20+ infiltration and decreased FOXP3+ and CD68+ infiltration, supporting an effector-leaning immune contexture rather than immune suppression.

#### 2.4.2. pTLS and Prognosis

In contrast to iTLS, the prognostic significance of pTLS appears less consistent across studies. Li et al. [49] concluded that high pTLS density was associated with improved OS/RFS, and that TLSs with GC morphology conferred even better outcomes. However, Zhang et al. [50] reported that pTLSs were associated with neutrophil infiltration and unfavorable prognosis. Another study [61] further revealed that TLSs located outside tumor nests or in altered microenvironmental niches may be dysfunctional and associated with immune suppression. There are several possible reasons for the discrepancy. Beyond the distance issue described above, several biological and methodological factors may explain why pTLS shows less consistent prognostic value than iTLS. First, pTLS density alone may mix immature lymphoid aggregates with functionally mature TLSs. In the high-pTLS density subgroup, GC-positive TLSs identified patients with better survival [49]. Therefore, studies that classify pTLSs only by presence, density or area may dilute the protective effect of mature pTLSs by combining them with early or poorly organized lymphoid aggregates. Second, the immune context surrounding pTLSs may modify or even reverse their prognostic implication. In the protective pattern described by Li et al. [49], high pTLS density was accompanied by higher CD3+, CD8+ and CD20+ infiltration and lower FOXP3+, CD68+ and PD-1+ infiltration, indicating an adaptive immune-enriched microenvironment. In contrast, Zhang et al. reported that peritumor TLSs were associated with CD15+ neutrophil infiltration and inferior prognosis, implying that some pTLS-rich regions may coexist with neutrophil-dominant or myeloid-skewed inflammation rather than productive antitumor immunity [50]. Its prognostic meaning likely depends on whether the surrounding niche is lymphocyte-dominant, GC-competent and immune-reactive or instead accompanied by suppressive myeloid/neutrophil programs. Third, cohort composition and quantification strategy may amplify these biological differences. A previous meta-analysis [55] found that pooled pTLS density was not significantly associated with OS or RFS in HCC. However, after excluding the Zhang et al. [50] cohort, high pTLS density became significantly associated with improved OS and RFS, with HRs of 0.42 and 0.45, respectively. The same analysis noted that pTLS results were unstable and potentially affected by differences in peritumoral sampling extent, tumor-stage distribution and cut-off definition [55]. This concern is supported by Sarelin et al. [51], who used three pTLS quantification methods and found that high pTLS density, maximum length and 5 mm hotspot count were all independently associated with improved 5-year OS in resected HCC, while combined grading of pTLSs and iTLSs further improved prognostic stratification. Therefore, for imaging-based studies, the pTLS should be modeled as a spatially and functionally heterogeneous compartment. Peritumoral ROIs and radiomics should be harmonized with the pathological pTLS definition, and sensitivity analyses using different ring widths may be necessary before linking pTLS-related biology to imaging phenotypes.

#### 2.4.3. TLS and Immunotherapy Response

For the assessment of therapeutic response, a TLS may evolve from a prognostic marker to a response predictor, with stronger spatial dependence. In neoadjuvant immune checkpoint blockade (ICB)-treated HCC, Shu et al. [36] found that TLS swere present in 63.2% of post-treatment tumors, and treatment significantly increased the intratumoral predominance of TLSs (iTLS proportion 72.9% vs. 54% in untreated controls). Critically, patients in the upper tertile of iTLS density exhibited 30-month RFS of 100%, compared with 38.5% in the middle and lower tertiles. The fundamental biological rationale is that the tumor microenvironment (TME) of high TLS density patients already exhibits a robust pre-existing immune response, characterized by abundant lymphocyte infiltration and efficient antigen presentation capabilities, as evidenced by references [36,60,62,63]. In contrast, patients with low TLS density typically display characteristics of “immune-cold tumors” and may necessitate more aggressive immune activation approaches—such as dual immune checkpoint blockade or combinations involving oncolytic viruses, tumor vaccines, and other modalities—with the goal of inducing the formation of new TLSs and thereby converting “cold” tumors into “hot” tumors.

Spatial analyses have provided further evidence that TLS-related immune activity is strongly dependent on anatomical location. High-density pTLS regions have been reported to contain mature B cells together with coordinated CXCL9/CXCL10 expression, suggesting that the tumor invasive margin may serve as an important site of immune activation [62]. However, pretreatment biopsy studies detected TLSs in only 12.5% of cases, whereas CD20-positive/CXCL13-positive lymphocyte aggregates were detected in 46.2% and were significantly associated with subsequent pathological response [36]. This observation suggests that biologically relevant immune organization may not always fulfill the strict morphological criteria for mature TLSs.

Under immunotherapy, iTLS may be a more actionable and predictable biological marker than pTLS. Shu et al. [36] explicitly demonstrated that iTLS density, rather than pTLS density, was a better predictor of pathological response and postsurgical RFS after neoadjuvant ICB. They also suggested that iTLS density and pathological response were among the strongest predictors of RFS. Furthermore, ICB appears to shift TLS patterns from peritumoral predominance toward intratumoral enrichment. pTLSs show a less consistent or even negligible prognostic association with survival in HCC cohorts, with varying results across studies.

### 2.5. TLSs Versus Ectopic Lymphoid Structures: Protective Immunity or Tumor-Promoting Niche?

Finkin et al. [20] proposed a framework based on the opposite concept that hepatic ectopic lymphoid-like structures (ELSs) can function as microniches for tumor progenitor cells and are associated with poor outcomes. This does not mean that ELSs are absent from HCC or that ELSs and TLSs are completely separate entities. In contrast, ELS is often used as a broader descriptive term for ectopic lymphoid aggregates arising in chronically inflamed non-lymphoid tissues, whereas TLS usually refers to more organized lymphoid structures that at least partially reproduce secondary lymphoid organ-like features, including B-cell follicles, T-cell zones, follicular dendritic cell networks, germinal-center reactions and/or HEVs [38,64]. Therefore, the distinction between ELSs and TLSs is not purely anatomical, but structural and functional. Immune-reactive TLSs are generally discussed as sites of local antigen presentation, B/T-cell cooperation and adaptive antitumor immunity, while the hepatic ELS described by Finkin et al. emphasized chronic injury-associated inflammatory niches that may support malignant hepatocyte progenitor cells before they acquire self-sustaining tumorigenic programs [20,38]. This distinction is particularly relevant in HCC, because chronic hepatitis, fibrosis, regeneration and immune tolerance frequently coexist in the background liver and at the tumor–liver interface. Thus, ELS-like structures may occur in chronically injured liver, peritumoral liver tissue and HCC-associated inflammatory niches. The key issue is not whether they are inside or outside the tumor, but whether a given lymphoid aggregate is organized toward productive antitumor immunity or toward chronic inflammation-associated regeneration and tumor promotion. This interpretation does not negate the favorable prognostic signal of mature iTLSs reported in HCC, nor the treatment-associated TLS remodeling observed after neoadjuvant immunotherapy [36]. Instead, it indicates that ectopic lymphoid aggregates in HCC should be subclassified according to spatial location, maturation state, cellular composition and functional program. In practical terms, GC-containing, HEV-associated and cytotoxic lymphocyte-enriched TLSs should be distinguished from poorly organized, chronic injury-associated ELS-like aggregates enriched in protumor inflammatory, regenerative or progenitor-cell signals. Overall, the biological impact of TLS-related structures in HCC is context dependent and may range from productive antitumor immunity to chronic inflammation-associated tumor promotion.

## 3. Conventional Assessment Methods of TLS and Its Limitations

Histopathology, such as immunohistochemistry (IHC), multiplex IHC (mIHC) and multiplex immunofluorescence (mIF), quantitative PCR (qPCR), spatial transcriptomics and proteomics, remains the gold standard for TLS structure, maturation and biology in HCC patients. However, the following limitations limits its application. First, the procedure is invasive, and selection bias is an issue. Only patients who undergo resection or biopsy provide TLS ground truth. However, tissue acquisition is difficult for unresectable or advanced HCC. Furthermore, the occurrence of TLSs is dynamic and can be remodeled by immunotherapy [58]. However, repeated sampling in HCC is limited by the risk of bleeding, infection and tumor seeding, as well as patient fitness, making longitudinal TLS monitoring clinically impractical. Much TLS evidence comes from cohorts that have undergone surgery, which limits its applicability to populations receiving systemic therapy. Second, sampling errors and scale mismatches cannot be avoided. A TLS is spatially heterogeneous and dependent on the tissue compartment. In a representative HCC cohort, iTLS was present in only 31.7% of cases, whereas pTLS was reported in 95% of cases [49]. This suggests that the absence of TLSs in tissue samples may be due to the location of the sample rather than the true absence of TLS biology at the lesion scale. This creates a scale mismatch. While pathology provides high-fidelity micro-architecture within sampled blocks, clinical decision-making implicitly requires a lesion-level immune phenotype. Third, cross-study comparability is weakened by non-uniform definitions and quantification. Differences in distance definitions for peritumoral TLS, maturity grading schemes and density cutoffs create heterogeneous TLS endpoints across studies. These differences can change the labeled phenotype and thereby the apparent association with outcome. Consequently, refining pathology alone cannot meet the practical requirements of oncology in the immunotherapy era, highlighting the importance of non-invasiveness, whole-tumor representation and longitudinal monitoring.

Multi-parameter imaging offers practical advantages over pathology by enabling longitudinal and whole-lesion assessment of the tumor–peritumoral unit. Furthermore, multiphasic and multiparametric acquisitions provide complementary macroscopic surrogates of perfusion-related microcirculation, microvascular organization, diffusion-derived cellularity proxies and susceptibility effects associated with hemorrhage [65,66,67]. Although indirect, these signals plausibly converge on biological processes that are crucial to TLSs, such as vascular niche formation, stromal remodeling and immune cell recruitment [7]. Accordingly, radiological imaging may help reduce sampling-driven label instability at the lesion level and support preoperative stratification. They could also provide a practical basis for longitudinal monitoring when tissue cannot be obtained repeatedly. With the aid of artificial intelligence, emerging HCC studies have revealed the capability of multi-parameter imaging for predicting TLS status, such as maturity, density, further for identifying immunotherapy benefit.

### The “Ground Truth” Dilemma: Pathology as an Imperfect Gold Standard

While histopathology remains the gold standard for TLS assessment, an important conceptual tension deserves explicit recognition: pathology itself is prone to sampling bias that creates systematic discordance with imaging. In a representative HCC cohort, iTLS was present in only 31.7% of cases when sampled, whereas pTLS was reported in 95% [49]. This disparity suggests that a “negative” pathology result may reflect sampling location rather than true biological absence—a phenomenon we term “scale mismatch”. Specifically, pathology provides high-fidelity micro-architecture within sampled blocks (millimeter scale), whereas clinical decision-making and imaging implicitly require a lesion-level immune phenotype (centimeter scale).

**Figure 2:**
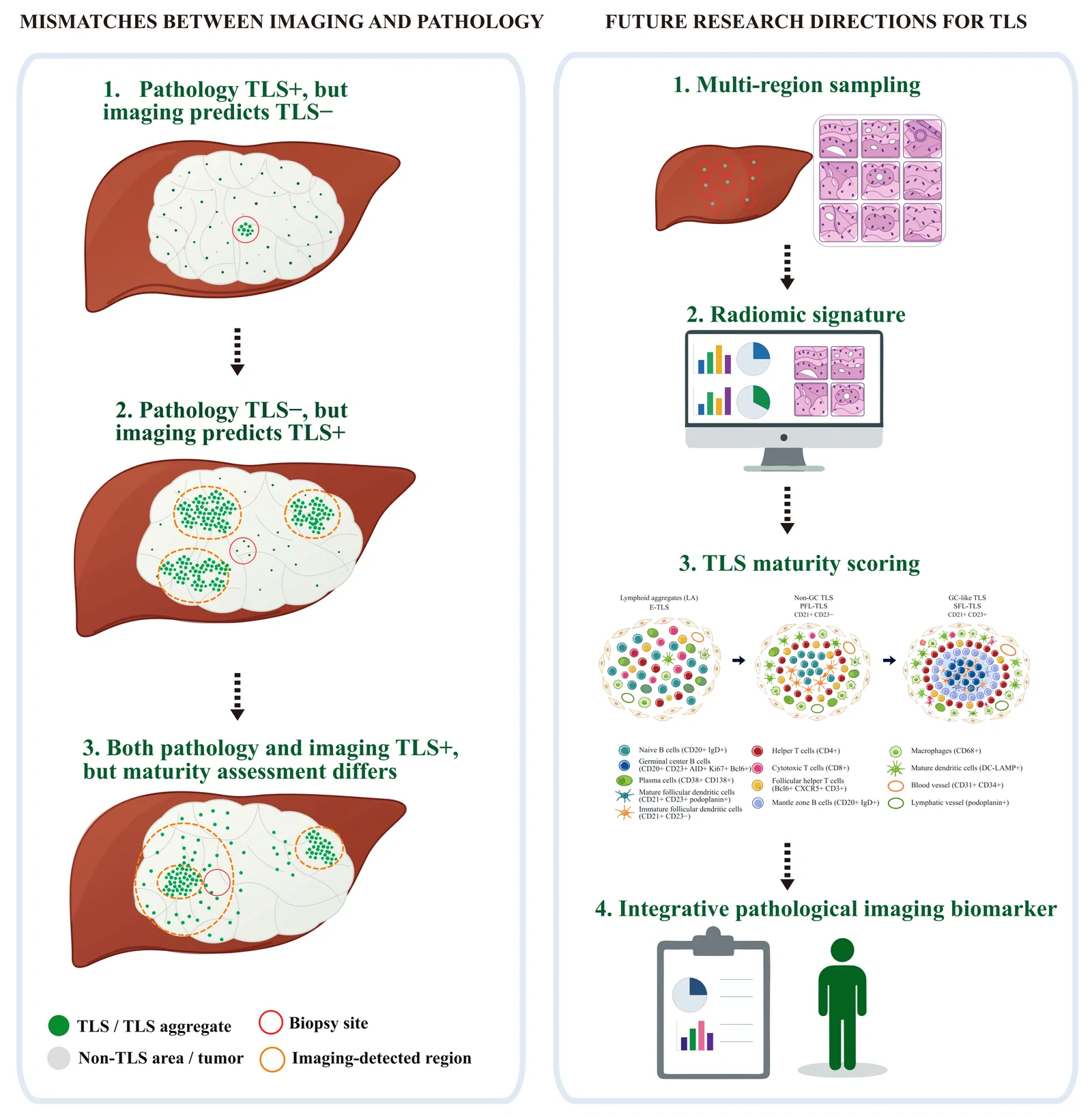
Mismatches between imaging and pathological findings of tertiary lymphoid structures (TLSs) in hepatocellular carcinoma, and future research directions for TLSs.

As illustrated in Figure 2, three common scenarios can lead to radiology–pathology discordance. First, a sampled region may contain TLSs, but the sampled tissue may not reflect the overall TLS burden of the tumor. Second, TLS-rich regions may be missed because they are located outside the sampled area. Third, TLS maturation may differ across spatial locations within the same lesion, such that a single tissue specimen cannot capture the full spectrum of TLS states. These scenarios highlight a fundamental scale mismatch: pathology provides high-resolution information at the tissue scale, whereas imaging evaluates the tumor and its surrounding tissue at the whole-lesion scale. These scenarios are not merely theoretical: in neoadjuvant immunotherapy studies, pretreatment biopsies detected TLSs in only 12.5% of cases, whereas CD20+CXCL13+ lymphocyte aggregates were detected in 46.2% [36], suggesting that conventional pathology may underreport TLS-related biology due to sampling limitations.

## 4. Current Applications of Integrated Multiparametric Imaging Techniques and Artificial Intelligence Algorithms in Non-Invasive Prediction of TLSs

### 4.1. Non-Invasive Assessment of TLS Structures in Hepatocellular Carcinoma Based on Contrast-Enhanced CT

Non-invasive assessment of TLSs in HCC through integrating preoperative multiphase contrast-enhanced CT has made significant strides. Through a retrospective single-center study involving 142 HCC patients, Li et al. [23] revealed that intratumoral arteries, intratumoral hemorrhage, hepatitis B/C status, elevated platelet counts (≥186.5 × 10^9^/L), and aspartate aminotransferase (AST ≥ 33.2 IU/L) served as predictors for the occurrence of iTLSs. The clinical-imaging nomogram also demonstrated robust discrimination in the training cohort and through five-fold cross-validation (AUC 0.79 and 0.75, respectively). A possible explanation is that intratumoral arteries and intratumoral hemorrhage were linked to tumor aggressiveness and immune evasion, potentially contributing to the absence of iTLSs. Notably, immune-high HCC exhibited significantly lower intratumor arterial vessel density, suggesting compromised arterial angiogenesis in the tumors of patients with immune-high HCC [68,69]. While this study highlighted the utility of CT semantic features, it was primarily limited by its reliance on qualitative imaging correlations rather than objective quantitative characterization.

Accordingly, artificial intelligence algorithms, such as radiomics analysis, have been employed to mine high-dimensional quantitative data, substantially enhancing diagnostic performance of the occurrence of iTLSs. A machine-learning model (RBF-SVM) based on arterial phase and venous phase CT images of 246 HCC patients from a multicenter retrospective study achieved superior AUCs of 0.93 and 0.90 in the training and testing sets, respectively, significantly outperforming conventional semantic models (AUC 0.79) [59]. Wu et al. [70] identified a combined nomogram including albumin–bilirubin (ALBI) grade (≥2) and radiomics score from preoperative CT images (arterial and portal venous phases), and this combined model achieved an improved validation AUC of 0.91, demonstrating a synergistic effect between clinical biomarkers and radiomic features.

However, prior research has been largely confined to iTLS assessment, neglecting its substantial spatial heterogeneity. With deepening investigation of the tumor microenvironment, pTLS, as a core immune structure at the tumor–host interface, is increasingly recognized for its independent prognostic value and synergistic effects with iTLS. A large-scale, multicenter retrospective study (*n* = 697) developed a combined model through integration of three selected clinical parameters (BCLC stage, serum AST level, and cirrhosis) with the multi-phase (AP, PVP, and DP) radiomics score from the composite image of intratumoral and 10 mm peritumoral regions; it achieved optimal predictive performance for TLS status assessment, with the highest AUC values of 0.83 (95% CI: 0.76–0.90) in the validation cohort and 0.83 (95% CI: 0.77–0.89) in the test cohort [71], underscoring the importance of considering both iTLS and pTLS in a comprehensive manner.

CT-based TLS assessment offers several advantages, including its non-invasive nature, its widespread clinical availability as a routine imaging modality for HCC, and the predictive value of semantic features such as intratumoral arteries and hemorrhage. The integration of artificial intelligence algorithms, particularly radiomics analysis, substantially enhances diagnostic performance, with machine-learning models achieving superior AUCs (0.90–0.93) compared to conventional semantic models [59]. Furthermore, combined nomograms incorporating clinical biomarkers (e.g., ALBI grade, BCLC stage, AST levels) with multiphase radiomics scores demonstrate synergistic effects and improved predictive accuracy. Additionally, composite imaging of intratumoral and peritumoral regions enables comprehensive evaluation of both iTLS and pTLS, addressing spatial heterogeneity. However, notable limitations persist: traditional semantic assessments suffer from inherent subjectivity and lack of standardization; most studies are single-center retrospective designs with insufficient external validation; prior research has been predominantly confined to iTLS assessment while neglecting pTLS; and the generalizability and clinical utility of AI-based models require further prospective, multicenter validation. Moreover, the absence of standardized definitions and grading systems for TLS assessment on CT hampers result comparability across studies.

### 4.2. Non-Invasive Assessment of TLS Structures in Hepatocellular Carcinoma Based on Multiparametric MRI

Compared to CT, magnetic resonance imaging (MRI) offers distinct advantages for hepatocellular carcinoma (HCC) assessment, characterized by superior soft-tissue resolution and a versatile range of functional sequences. These capabilities facilitate the multidimensional quantification and visualization of tertiary lymphoid structure (TLS)-related pathomolecular features—encompassing morphological, structural, functional, and metabolic perspectives—thereby providing a robust, non-invasive framework for assessing tumor invasiveness and predicting therapeutic response [72,73].

#### 4.2.1. Multiphase Contrast-Enhanced MRI

Multiphase contrast-enhanced MRI integrates the advantages of MRI, including multi-parametric imaging and superior soft tissue contrast, with the use of intravenous contrast agents. By acquiring sequential scans at multiple phases, multiphase contrast-enhanced MRI enables the characterization of hemodynamic kinetics, providing insights into underlying tumor vascularity and oxygenation—key factors that influence lymphocyte infiltration and the formation of TLSs [73,74]. Long et al. [60] conducted a large-scale, multicenter study (*n* = 660) to systematically evaluate the utility of a transfer learning radiomics (TLR) model with multiphase MRI in predicting iTLS status. The TLR model demonstrated high discriminative power, achieving AUCs of 0.91, 0.85, and 0.85 in the training, internal, and external validation cohorts, respectively. This performance significantly outperformed both standalone radiomics (AUC = 0.71) and isolated transfer learning architectures (AUC = 0.78). Habitat imaging represents an innovative methodology for quantifying intratumoral heterogeneity by segmenting tumors into physiologically distinct subregions based on voxel-level functional imaging data [75,76]. A retrospective single-center study with 334 HCC patients has developed a hybrid model that combines DCE-MRI-derived habitat features with clinical variables (AFP and hepatitis virus infection) and conventional radiomics features to preoperatively identify iTLS status; this hybrid model achieved the highest discrimination (AUC = 0.83 and 0.84 in the training and test sets, respectively), significantly outperforming the clinical, radiomics, and clinical–radiomics models in both sets, indicate that incorporating habitat-derived features can further enhance model performance for TLS status identification [77]. However, limited studies have evaluated TLS maturation status based on multiparametric MRI. Future large-scale, prospective, multi-center studies are necessary to confirm the robustness and clinical utility of TLS maturation status assessment with multiparametric MRI.

#### 4.2.2. Gadoxetic Acid-Enhanced MRI Hybrid Prediction Models

Gadoxetic acid (Gd-EOB-DTPA)-enhanced MRI, characterized by its unique hepatocyte-specific uptake mechanism, provides indispensable value for the precision diagnosis of hepatocellular carcinoma (HCC). Li et al. [78] comprehensively mined radiomics features from Gd-EOB-DTPA MRI image of 334 HCC patients at two tertiary hospitals, and integrated them with critical clinical–radiological (CR) factors, including BCLC stage, intratumoral hemorrhage, and the presence of satellite nodules, to construct a multidimensional hybrid prediction model. This hybrid model achieved significantly superior AUCs of 0.823 (95% CI: 0.728, 0.895) and 0.875 (95% CI: 0.725, 0.960) in the internal and external validation cohorts, respectively. Among patients receiving postoperative targeted therapy combined with immunotherapy, the model demonstrated robust prognostic stratification capabilities: the high-risk group identified by the model derived substantial survival benefits from the combination therapy, whereas improvement was limited in the low-risk group. These findings robustly validate the clinical utility and stability of the model in guiding personalized treatment strategies.

#### 4.2.3. Susceptibility-Weighted Imaging-Based Model

Previous studies [23,78,79] have confirmed intratumoral hemorrhage as a key predictor of iTLSs in HCC patients. The underlying mechanism involves erythrocyte lysis following hemorrhage, releasing heme, iron metabolites, and damage-associated molecular patterns (DAMPs), which reshape the tumor immune microenvironment and thereby regulate TLS formation and development. Susceptibility-weighted imaging (SWI), as a technique utilizing endogenous magnetic susceptibility differences for imaging, exhibits extreme sensitivity to hemoglobin degradation products, iron deposition, and microhemorrhagic foci, providing a unique perspective for non-invasive assessment of tumor microvascular characteristics and microhemorrhage, suggesting substantial potential in iTLS prediction [80]. However, conventional qualitative SWI assessment remains limited by subjective bias and difficulty in identifying extremely subtle hemorrhagic foci. Artificial intelligence algorithms, through excavation of high-dimensional quantitative features from image data, can effectively overcome visual assessment limitations, enabling objective and precise identification of iTLS-related imaging biomarkers. Liu et al. [63] determined random forest (RF) as the optimal classifier for iTLS prediction (independent validation set AUC = 0.771) through comparison of five mainstream machine-learning algorithms. To further deconstruct the model’s “black box” attributes, the study introduced the SHAP (Shapley additive explanations) interpretability framework, and this analysis revealed that “log-sigma first-order minimum” indirectly reflected intratumoral blood supply homogeneity through quantification of signal extremes, while “exponential gray level size zone matrix zone entropy” precisely captured imaging signal heterogeneity resulting from microvascular remodeling. Survival analysis further confirmed that the iTLS-positive group constructed based on the SWI model demonstrated significant survival benefit when receiving TKI-ICI combined therapy (*p* < 0.05). This study not only provides interpretable quantitative evidence for iTLS assessment but also offers a simple, efficient digital solution for HCC individualized decision-making in resource-limited settings.

#### 4.2.4. Intravoxel Incoherent Motion Diffusion-Weighted Imaging Multiparametric Fusion Models

Intravoxel incoherent motion diffusion-weighted imaging (IVIM-DWI), through bi-exponential modeling, can effectively distinguish pure water molecular diffusion from microcirculatory perfusion effects. Ma et al. [81] confirmed in a prospective study with 168 HCC patients that a combined radiomics score (Rad-score) model integrating all IVIM parametric maps (including true diffusion coefficient Dt, pseudo-diffusion coefficient Dp, perfusion fraction f, and apparent diffusion coefficient ADC) achieved significantly superior performance compared to single-parameter models. Further fusion with radiomics scores, ADC_90Percentile, f_Maximum, satellite nodules and lymphocyte count resulted in a multimodal model achieving an AUC of 0.86 to identify the maturation stage of the TLSs in the independent test set. The incidence of vessels encapsulating tumor clusters (VETC) was found to be lower in the iTLS+ group than in the iTLS- group [23]. Additionally, the VETC-positive group exhibited a significantly higher microvessel density within the tumor compared to the VETC-negative group [82]. ADC is a calculated value that incorporates data regarding Dt and f. This observation might account for the lower f_Maximum value and ADC_90Percentile observed in the TLSs+ group as opposed to the TLSs- group. This study successfully achieved synchronous deconstruction and evaluation of multi-dimensional biological information including HCC cellularity, vascularization degree, and immune microenvironment.

Integrated multimodal MRI (including conventional multiphase enhancement, SWI, and IVIM functional imaging) and deep-learning models have emerged as powerful tools for non-invasive assessment of HCC immune microenvironment, particularly for predicting TLS status. By excavating high-dimensional spatial features imperceptible to the naked eye, these models successfully bridge macroscopic imaging manifestations with microscopic immune phenotypes, demonstrating not only excellent stability in patient prognosis prediction but also substantial clinical application potential in screening immune checkpoint inhibitor (ICI) beneficiaries.

### 4.3. Complementary Roles of Intratumoral and Peritumoral Imaging Features

This spatial–dimensional biological difference of iTLS and pTLS determines that imaging features based on single regions cannot comprehensively characterize the complex immune landscape associated with TLSs. Long et al. [62] extended the research scope to include the peritumoral region, providing a comprehensive dissection of the immunobiological landscape of peritumoral TLSs (pTLSs). Their study confirmed that high-density pTLS regions are enriched with mature B-cell infiltrates; clinically, pTLS-high patients showed significantly prolonged median OS (*p* < 0.05) and enhanced immunotherapy responsiveness (*p* = 0.030). Through spatial pseudo-time analysis and immunohistochemical validation, the researchers further demonstrated that pTLS development in HCC is closely associated with the co-expression of CXCL9 and CXCL10 chemokines. Based on this molecular mechanism, the team developed an integrated “intratumoral–peritumoral” radiomics (mPI) model, which achieved excellent performance for predicting pTLS density in external validation (AUC 0.91). Critically, the radiomics scores positively correlated with CXCL9/CXCL10 expression levels, thereby imbuing the radiomic features with explicit immunobiological significance. Particularly importantly, stability validation across different centers and cross-field strength devices (1.5T/3.0T MRI) (*p* = 0.61–0.89) demonstrated that the intratumoral–peritumoral combined strategy has potential for clinical use.

## 5. Clinical Application Value of Imaging-Based TLS Prediction Model

### 5.1. Prognostic Stratification Value of TLS Prediction Model

TLS prediction models not only provide non-invasive, longitudinally monitorable digital biomarkers for evaluating TLSs in HCC patients but also serve as effective tools for prognostic stratification. The high risk of iTLS abundance, as defined by the RBF-SVM model, was negatively correlated with recurrence-free survival (RFS) and overall survival (OS) [59]. Li et al. [23] further elucidated that intratumoral hemorrhage, as a TLS-negative predictor, was associated with vascular dysfunction and extracellular matrix degradation; its imaging features can serve as auxiliary indicators for precise identification of patient subgroups with high recurrence tendency. Long et al. [60] confirmed that the iTLS-positive group predicted by the TLR model demonstrated significant survival advantages in the external validation cohort, with both RFS (HR = 0.64, 95%CI: 0.48–0.85, *p* = 0.001) and OS (HR = 0.66, 95%CI: 0.48–0.90, *p* = 0.007) significantly prolonged. Multivariate analysis further established the independent prognostic value of this model, with its efficacy unaffected by traditional pathological factors including Barcelona Clinic Liver Cancer (BCLC) staging, microvascular invasion (MVI), and tumor differentiation degree, suggesting that radiomic TLS assessment can serve as an important complementary branch for postoperative risk stratification. In the pTLS prediction domain, the mPI model constructed by Long et al. [62] similarly demonstrated excellent prognostic stratification capability. Kaplan–Meier analysis indicated that in training, temporal, and external validation cohorts, the high pTLS density group identified by the model exhibited significant OS (HR 0.20–0.44, *p* = 0.005 to <0.001) and RFS (HR 0.25–0.73, *p* = 0.015 to <0.001) benefits. Multivariate Cox regression analysis further confirmed that mPI model score was a powerful independent predictor of RFS (HR = 0.56, 95%CI: 0.46–0.69, *p* < 0.001) and OS (HR = 0.51, 95%CI: 0.40–0.66, *p* < 0.001), with prognostic assessment efficacy significantly surpassing traditional imaging features and clinical biochemical indicators. Meanwhile, the hybrid model in Li’s study [78], based on Gd-EOB-DTPA-enhanced MRI, demonstrated that the high TLS score group predicted by the model showed high concordance with pathologically confirmed TLS-positive status. This group’s 1-year, 2-year, and 3-year disease-free survival (DFS) rates were 78.5%, 56.3%, and 42.1%, respectively, with significantly superior prognosis compared to the low-risk group. Hence, these TLS prediction models, leveraging radiomics and hybrid imaging techniques, offer promising non-invasive tools for accurate prognostic stratification in HCC patients, potentially enhancing personalized treatment strategies and improving patient outcomes.

### 5.2. Immunotherapy Benefit Prediction Based on TLS Model

Addressing the current clinical bottleneck of low objective response rates (ORRs) (only 11–30%) in HCC first-line combined therapy, precise screening of potential beneficiaries has become critical for efficacy improvement [36]. TLS prediction models based on multiparametric imaging demonstrate profound clinical translational significance in guiding decision-making for immunotherapy.

Long et al. [60], based on the TLR model, prospectively evaluated 101 advanced HCC patients receiving first-line immune checkpoint inhibitor combined with anti-angiogenic agent (TKI-ICI) therapy. Results indicated that the iTLS-positive group determined by the model achieved an ORR of 36%, significantly superior to the negative group, accompanied by significantly prolonged OS (HR = 0.52, 95%CI: 0.29–0.91, *p* = 0.02) and PFS (HR = 0.35, 95%CI: 0.20–0.62, *p* < 0.001). This study further utilized the Cancer Genome Atlas (TCGA) database for pathway enrichment analysis, confirming that high TLR scores were highly correlated with inflammatory response, adaptive immune response, and oncogenic signaling pathway activation (including IL-6-JAK-STAT3, TNFα-NFκB, mTORC1). This finding not only establishes imaging features as molecular anchors for immunotherapy response but also provides robust mechanistic support for the biological association between imaging phenotypes and tumor microenvironment remodeling. In the pTLS prediction domain, the mPI model further confirmed the unique value of spatial heterogeneity features in predicting immunotherapy benefits [62]. Among advanced HCC patients receiving TKI-ICI therapy, the high pTLS density group identified by this model demonstrated a superior objective response rate (ORR 43.7% vs. negative group, *p* = 0.03). Particularly importantly, mPI scores showed significant positive correlation with CXCL9/CXCL10 H-scores and specific TLS gene signatures (including TLS-50, 12-chemokine signature) (*p* < 0.001). This finding demonstrates that TLS prediction models can precisely capture key biological processes of chemokine-mediated lymphocyte recruitment and TLS ectopic development. For postoperative adjuvant therapy indication selection, among 72 postoperative adjuvant therapy patients, this model achieved precise efficacy stratification: only the patients with high TLS risk identified by the hybrid model based on Gd-EOB-DTPA-enhanced MRI in Li’s study [78] derived significant survival benefits from adjuvant immunotherapy (DFS: *p* < 0.05). Analysis after propensity score matching (PSM) correction further elucidated that TLS-positive status was not only a favorable prognostic indicator but also exhibited synergistic efficacy enhancement with immunotherapy, providing important decision-making foundations for individualized postoperative adjuvant regimen formulation in HCC. Therefore, TLS prediction models can provide critical decision-making support for selection of the above individualized treatment strategies. However, the translational pathway and exact efficacy of the prediction model in clinical practice require further verification and closed-loop assessment in future prospective study. Table 1 shows integrated multiparametric imaging techniques and artificial intelligence algorithms for the diagnosis of TLSs.

## 6. Challenges and Future Directions

Despite rapid progress in the non-invasive imaging assessment of TLSs in HCC, current imaging-based prediction models remain at an early stage of clinical translation. Most available studies are retrospective and focus on single-task classification, typically predicting the presence or absence of TLSs. However, a TLS is a spatially heterogeneous and dynamically remodeled immune structure, and its biological significance depends on maturation state, anatomical location, cellular composition, and treatment context. Consequently, high predictive performance alone does not necessarily indicate that an imaging model has captured TLS biology. Figure 2 summarizes the major patterns of radiology–pathology discordance and outlines potential directions for future research.

### 6.1. Improving the Granularity of TLS Labels

The first challenge is label granularity. Most current HCC imaging models are trained using binary or semi-quantitative pathological endpoints rather than biologically stratified TLS states [23,62,70,81]. However, a TLS is not a single biological state. Pathologically, TLSs can be staged from early lymphoid aggregates to primary follicle-like TLSs and secondary follicle-like TLSs, and previous HCC meta-analytic evidence suggests that simple TLS presence may not fully capture prognostic heterogeneity [55]. Mechanistically, TLS maturity, spatial embedding, immune-cell composition and treatment-related remodeling can all modify its prognostic and predictive meaning [39]. In addition, neoadjuvant immunotherapy in HCC can induce non-canonical involuted TLS morphology, characterized by B-follicle dispersion but persistent T-cell zones, mature DC-T-cell interactions and memory-associated signatures [36]. Consequently, a model trained on a coarse TLS label may achieve apparently high discrimination while remaining biologically under-specified, because the learned imaging signal may reflect a mixture of TLS location, density, maturation, stromal remodeling, vascular phenotype and treatment-related change rather than TLS presence alone. This does not invalidate current radiomic studies. It indicates that future models should move from single-task TLS detection toward multi-task or ordinal endpoints integrating TLS location, maturation and immune context. Emerging MRI work that directly attempts to predict iTLS maturity rather than simple iTLS presence is an important step in this direction, but spatially matched validation by multiplex pathology or spatial omics is still required before imaging features can be interpreted as specific surrogates of TLS maturity or function [23,36,39].

### 6.2. Establishing Spatially Matched Ground Truth

A second major challenge is the reliability of pathological labels used as the reference standard for imaging-based TLS prediction. Although histopathology provides high-resolution information on TLS morphology and cellular composition, tissue sampling may not adequately represent the whole tumor because TLSs are spatially heterogeneous. This creates a potential mismatch between millimeter-scale pathological sampling and the lesion-level information captured by CT or MRI.

As discussed above, iTLSs and pTLSs may have markedly different prevalence and prognostic implications, and the detection of TLSs can depend strongly on the location and extent of tissue sampling [49,50,51,52]. A negative pathological sample may therefore indicate that TLSs were not present in the sampled region rather than that the entire tumor lacks TLS-related biology. This issue is particularly important for artificial intelligence models because sampling-related label noise may be propagated directly into model training.

Further research is needed to establish spatially matched radiology–pathology datasets in which imaging regions of interest are linked as precisely as possible to corresponding tissue samples. Image-guided targeted sampling could be used to obtain tissue from radiologically defined regions of interest. Prospective, multicenter studies involving independent pathological review and repeated multi-region sampling will be essential to determine whether an imaging model predicts the whole-tumor TLS burden, the presence of mature TLSs, or merely the probability of detecting TLSs in a particular pathological sampling site. In this framework, imaging could serve not only as a predictive tool but also as a spatial prior for targeted tissue acquisition.

### 6.3. Improving Generalizability and Reproducibility

Another challenge is the generalizability of findings. Quantitative parameters are sensitive to factors such as scanner vendor, reconstruction kernel, slice thickness, contrast timing and acquisition parameters. External validation may still be protocol-coupled if sites share acquisition conventions, patient etiologies or regional practice patterns. For example, multicenter MRI radiomics studies reporting AUC values of around 0.82–0.88 in validation cohorts are encouraging. However, the key question is whether these models remain calibrated and discriminative under genuinely heterogeneous acquisition regimes and patient populations [78].

Subsequent studies will therefore prioritize rigorous multicenter and prospective validation. Standardized imaging acquisition protocols, harmonized preprocessing pipelines, clearly defined regions of interest, and transparent reporting of model development procedures should be encouraged. In addition, models should be evaluated under clinically relevant distribution shifts, including differences in scanner platforms, field strength, contrast agents, reconstruction settings, and patient characteristics. External validation should therefore be considered an essential component of model development rather than an optional final step.

### 6.4. Moving from Model Interpretability to Biological Interpretability

Interpretability is another important barrier to clinical translation. Many current imaging models use SHAP, feature-importance analysis, attention maps, or related techniques to explain model predictions. These methods can identify which imaging features contribute most strongly to a model’s output, but they do not necessarily explain the biological mechanisms that generate those imaging patterns.

This distinction is particularly important for TLSs because TLSs are dynamic structures whose morphology and composition may change during treatment. An imaging feature associated with TLS prediction at baseline may reflect not only lymphoid organization but also stromal remodeling, vascular changes, fibrosis, hemorrhage, or other components of the tumor microenvironment [36]. Therefore, identifying a predictive feature does not establish that the feature is biologically specific to TLSs.

Further research is warranted to move from post hoc feature attribution toward biologically testable hypotheses. Imaging features should be evaluated against spatially matched pathological and molecular measurements to determine whether specific imaging patterns correspond to TLS density, maturation, immune-cell composition, chemokine activity, vascular organization, or other biological processes. Such cross-scale validation would help distinguish statistical association from biological specificity and would improve the interpretability of imaging-derived TLS biomarkers.

### 6.5. Integrating Multimodal Imaging with Spatial Omics

Because no single imaging modality can directly visualize the full cellular and molecular architecture of TLSs, multimodal integration represents an important future direction. CT and MRI provide complementary information regarding tumor morphology, perfusion, diffusion, susceptibility, and tissue composition, whereas pathology and spatial omics can provide high-resolution information on cellular organization and molecular programs.

Rather than simply concatenating imaging and molecular features, in the future, studies should develop biologically informed hierarchical fusion strategies. In this framework, multiparametric imaging could provide a whole-lesion spatial map, while multiplex pathology and spatial transcriptomics could characterize the cellular and molecular features underlying selected imaging phenotypes.

Recent work provides a useful blueprint for this approach. A multicenter pTLS MRI study linked radiomics signatures with spatial transcriptomics and large RNA-sequencing resources and found that radiomic features associated with pTLS density were correlated with immune-regulatory programs, with an external validation AUC of 0.91 [62]. This type of cross-scale integration may reduce label noise, strengthen biological interpretation, and help determine whether imaging-derived TLS signatures remain meaningful across tumors with different TLS compositions and maturation states.

### 6.6. From Prediction to Prospective Clinical Validation

Ultimately, the value of imaging-based TLS prediction should be determined not only by discrimination metrics but also by whether the models improve clinical decision-making. Most current studies remain retrospective and focus primarily on predicting TLS status, prognosis, or treatment response. Prospective evidence demonstrating that TLS imaging biomarkers can alter treatment selection or improve patient outcomes remains limited.

Future work will therefore move toward prospective, multicenter, and decision-oriented validation. Rather than evaluating whether an imaging model simply predicts TLSs, studies should determine whether its use can improve clinically relevant decisions, such as identifying patients who are more likely to benefit from immunotherapy, selecting patients for intensified treatment, or identifying individuals who may require additional immune-modulating strategies.

Such trials should incorporate standardized imaging acquisition, predefined model thresholds, external validation, clinically meaningful endpoints, and appropriate calibration analyses. Importantly, treatment-specific validation will be necessary because TLS biology may change during immunotherapy and other systemic treatments [36]. Longitudinal imaging may therefore provide additional value by monitoring treatment-associated remodeling of TLS-related immune phenotypes rather than providing only a single pretreatment classification.

Overall, future development of imaging-based TLS assessment should proceed from simple binary prediction toward biologically informed, spatially resolved, and clinically validated immune phenotyping. A potential framework is to integrate multiparametric imaging as a whole-lesion spatial prior, spatial pathology and spatial omics as the biological validation layer, and prospective multicenter studies as the clinical validation layer. Such a framework may ultimately transform imaging-derived TLS biomarkers from exploratory associations into robust and biologically interpretable tools for precision immunotherapy in HCC.

## 7. Conclusions

Non-invasive profiling of TLSs is an effective method to support immune phenotyping in HCC, by extracting quantitative descriptors from routine multiparametric imaging that may reflect aspects of the intratumoral and peritumoral microenvironments. Furthermore, it holds the potential to function as a scalable pre-treatment screening tool, thereby enhancing patient prognostic stratification and facilitating personalized and precise treatment strategies. However, its dynamic and spatially heterogeneous nature, and its lack of generalizable interpretability, limits the clinical transformation of the prediction model. To overcome these challenges, future studies should focus on standardized TLS definitions, spatially matched imaging–pathology validation, multimodal imaging–omics integration, and prospective multicenter validation. Ultimately, imaging-based TLS assessment may help move from simple TLS detection toward non-invasive characterization of the tumor immune microenvironment.

## Figures and Tables

**Table 1:** Integrated multiparametric imaging techniques and artificial intelligence algorithms for the diagnosis of TLSs.

	Imaging Modality	Imaging Features of TLSs	Sensitivity (%)	Specificity (%)	Main Advantages	Main Disadvantages
CT	Multi-phase CT imaging and clinical features-based nomogram [23]	Intratumoral artery, intratumoral hemorrhage	82	53	Comprehensive tumor evaluation	Without quantitative analysis
	Multi-phase CT radiomics classifier [59]	Radiomic features from arterial and portal venous phases	-	-	Predicts RFS/OS non-invasively; captures tumor microenvironment	Limited to liver transplant cohort
	Dual-phase CT radiomics–clinical fusion model [70]	Radiomic features from arterial and portal venous phases	-	-	Machine-learning model integrating contrast-enhanced CT radiomics and clinical parameters	Single-center retrospective design
	Multi-region multi-phase CT radiomics fusion model [71]	Intratumoral + multi-region peritumoral radiomic features	-	-	Predicts pTLS; correlates with immunotherapy response	Manual ROI; imbalanced dataset
MRI	Multi-center MRI-based transfer learning radiomics (TLR) model [60]	Deep-learning features + handcrafted radiomic features	83–97	68–84	Predicts combination therapy response	Small immunotherapy cohort (*n* = 101)
	DCE-MRI habitat radiomics–clinical hybrid model [77]	Tumor habitats with distinct enhancement patterns + habitat-derived features	75–77	70–77	Captures intratumoral spatial heterogeneity	Single-center; TLS maturity not assessed
	Multi-center Gd-EOB-DTPA-enhanced MRI radiomics–clinical hybrid model with nomogram [78]	Multiparametric MRI radiomic features + clinical-radiological variables	62–94	76–93	Identifies immunotherapy beneficiaries	Expensive contrast; small external cohort (*n* = 37)
	SWI radiomics model [63]	Microhemorrhages, iron/hemoglobin deposits	79–80	68–75	Correlates with immune microenvironment remodeling in iTLS formation	Single-center; limited to hemorrhagic TLS
	Prospective IVIM-MRI radiomics–clinical fusion model [81]	True molecular diffusion and blood microcirculation perfusion	88–93	67–80	Simultaneously assess true molecular diffusion and microcirculation perfusion	Only IVIM sequence was used without incorporating multiparametric MRI
	Multi-region multi-phase MRI radiomics fusion model [62]	Intratumoral + peritumoral radiomic features	86–91	84–95	Multicenter study for predicting peritumoral TLS density in HCC	Manual segmentation

## Data Availability

No new data were created or analyzed in this study. Data sharing is not applicable to this article.

## References

[1] Sung H., Ferlay J., Siegel R.L., Laversanne M., Soerjomataram I., Jemal A., Bray F. (2021). Global Cancer Statistics 2020: GLOBOCAN Estimates of Incidence and Mortality Worldwide for 36 Cancers in 185 Countries. CA Cancer J. Clin..

[2] Ascari S., Chen R., De Sinno A., Stefanini B., Cescon M., Serenari M., Mosconi C., Tovoli F. (2026). Post-immunotherapy second-line strategies for hepatocellular carcinoma: State of the art and ongoing trials. World J. Gastroenterol..

[3] Robinson M.W., Harmon C., O’Farrelly C. (2016). Liver immunology and its role in inflammation and homeostasis. Cell. Mol. Immunol..

[4] Zong L., Peng H., Sun C., Li F., Zheng M., Chen Y., Wei H., Sun R., Tian Z. (2019). Breakdown of adaptive immunotolerance induces hepatocellular carcinoma in HBsAg-tg mice. Nat. Commun..

[5] Jia W., Yao Q., Wang Y., Mao Z., Zhang T., Li J., Nie Y., Lei X., Shi W., Song W. (2022). Protective effect of tertiary lymphoid structures against hepatocellular carcinoma: New findings from a genetic perspective. Front. Immunol..

[6] Zhang Y., Xu M., Ren Y., Ba Y., Liu S., Zuo A., Xu H., Weng S., Han X., Liu Z. (2024). Tertiary lymphoid structural heterogeneity determines tumour immunity and prospects for clinical application. Mol. Cancer.

[7] Teillaud J.L., Houel A., Panouillot M., Riffard C., Dieu-Nosjean M.C. (2024). Tertiary lymphoid structures in anticancer immunity. Nat. Rev. Cancer.

[8] Calderaro J., Petitprez F., Becht E., Laurent A., Hirsch T.Z., Rousseau B., Luciani A., Amaddeo G., Derman J., Charpy C. (2019). Intra-tumoral tertiary lymphoid structures are associated with a low risk of early recurrence of hepatocellular carcinoma. J. Hepatol..

[9] Yang M., Che Y., Li K., Fang Z., Li S., Wang M., Zhang Y., Xu Z., Luo L., Wu C. (2023). Detection and quantitative analysis of tumor-associated tertiary lymphoid structures. J. Zhejiang Univ. Sci. B.

[10] Munoz-Erazo L., Rhodes J.L., Marion V.C., Kemp R.A. (2020). Tertiary lymphoid structures in cancer—Considerations for patient prognosis. Cell. Mol. Immunol..

[11] Schumacher T.N., Thommen D.S. (2022). Tertiary lymphoid structures in cancer. Science.

[12] Sautès-Fridman C., Petitprez F., Calderaro J., Fridman W.H. (2019). Tertiary lymphoid structures in the era of cancer immunotherapy. Nat. Rev. Cancer.

[13] Dieu-Nosjean M.C., Goc J., Giraldo N.A., Sautès-Fridman C., Fridman W.H. (2014). Tertiary lymphoid structures in cancer and beyond. Trends Immunol..

[14] Nerviani A., Pitzalis C. (2018). Role of chemokines in ectopic lymphoid structures formation in autoimmunity and cancer. J. Leukoc. Biol..

[15] Jones G.W., Hill D.G., Jones S.A. (2016). Understanding Immune Cells in Tertiary Lymphoid Organ Development: It Is All Starting to Come Together. Front. Immunol..

[16] Asam S., Nayar S., Gardner D., Barone F. (2021). Stromal cells in tertiary lymphoid structures: Architects of autoimmunity. Immunol. Rev..

[17] Guillaume S.M., Beccaria C.G., Iannacone M., Linterman M.A. (2025). Tertiary Lymphoid Structures Across Organs: Context, Composition, and Clinical Levers. Immunol. Rev..

[18] Zhou J., Wang G., Chen Y., Wang H., Hua Y., Cai Z. (2019). Immunogenic cell death in cancer therapy: Present and emerging inducers. J. Cell. Mol. Med..

[19] Deng S., Chen Y., Song B., Wang H., Huang S., Wu K., Chu Q. (2025). Tertiary lymphoid structures in cancer: Spatiotemporal heterogeneity, immune orchestration, and translational opportunities. J. Hematol. Oncol..

[20] Finkin S., Yuan D., Stein I., Taniguchi K., Weber A., Unger K., Browning J.L., Goossens N., Nakagawa S., Gunasekaran G. (2015). Ectopic lymphoid structures function as microniches for tumor progenitor cells in hepatocellular carcinoma. Nat. Immunol..

[21] Boettler T., Gill U.S., Allweiss L., Pollicino T., Tavis J.E., Zoulim F. (2022). Assessing immunological and virological responses in the liver: Implications for the cure of chronic hepatitis B virus infection. JHEP Rep..

[22] Mihm S. (2018). Danger-Associated Molecular Patterns (DAMPs): Molecular Triggers for Sterile Inflammation in the Liver. Int. J. Mol. Sci..

[23] Li P., Liang Y., Zeng B., Yang G., Zhu C., Zhao K., Xu Z., Wang G., Han C., Ye H. (2022). Preoperative prediction of intra-tumoral tertiary lymphoid structures based on CT in hepatocellular cancer. Eur. J. Radiol..

[24] Tang H., Zhu M., Qiao J., Fu Y.X. (2017). Lymphotoxin signalling in tertiary lymphoid structures and immunotherapy. Cell. Mol. Immunol..

[25] Vondenhoff M.F., Greuter M., Goverse G., Elewaut D., Dewint P., Ware C.F., Hoorweg K., Kraal G., Mebius R.E. (2009). LTbetaR signaling induces cytokine expression and up-regulates lymphangiogenic factors in lymph node anlagen. J. Immunol..

[26] Barone F., Gardner D.H., Nayar S., Steinthal N., Buckley C.D., Luther S.A. (2016). Stromal Fibroblasts in Tertiary Lymphoid Structures: A Novel Target in Chronic Inflammation. Front. Immunol..

[27] Ruddell R.G., Knight B., Tirnitz-Parker J.E., Akhurst B., Summerville L., Subramaniam V.N., Olynyk J.K., Ramm G.A. (2009). Lymphotoxin-beta receptor signaling regulates hepatic stellate cell function and wound healing in a murine model of chronic liver injury. Hepatology.

[28] Cannella R., Ronot M., Sartoris R., Cauchy F., Hobeika C., Beaufrere A., Trapani L., Paradis V., Bouattour M., Bonvalet F. (2021). Enhancing capsule in hepatocellular carcinoma: Intra-individual comparison between CT and MRI with extracellular contrast agent. Diagn. Interv. Imaging.

[29] Nishioka E., Sofue K., Maruyama K., Ueshima E., Ueno Y., Tsurusaki M., Komatsu S., Fukumoto T., Murakami T. (2023). Improved diagnosis of histological capsule in hepatocallular carcinoma by using nonenhancing capsule appearance in addition to enhancing capsule appearance in gadoxetic acid-enhanced MRI. Sci. Rep..

[30] Ueshima E., Sofue K., Kodama T., Yamamoto S., Komatsu M., Komatsu S., Ishihara N., Umeno A., Yamaguchi T., Hori M. (2025). Gadoxetic Acid-Enhanced Magnetic Resonance Imaging Features Can Predict Immune-Excluded Phenotype of Hepatocellular Carcinoma. Liver Cancer.

[31] Xie X.Y., Chen R. (2025). Research progress of MRI-based radiomics in hepatocellular carcinoma. Front. Oncol..

[32] Pereira J.P., Kelly L.M., Cyster J.G. (2010). Finding the right niche: B-cell migration in the early phases of T-dependent antibody responses. Int. Immunol..

[33] Hong W., Yang B., He Q., Wang J., Weng Q. (2022). New Insights of CCR7 Signaling in Dendritic Cell Migration and Inflammatory Diseases. Front. Pharmacol..

[34] Siliņa K., Soltermann A., Attar F.M., Casanova R., Uckeley Z.M., Thut H., Wandres M., Isajevs S., Cheng P., Curioni-Fontecedro A. (2018). Germinal Centers Determine the Prognostic Relevance of Tertiary Lymphoid Structures and Are Impaired by Corticosteroids in Lung Squamous Cell Carcinoma. Cancer Res..

[35] Chen Y., Wu Y., Yan G., Zhang G. (2024). Tertiary lymphoid structures in cancer: Maturation and induction. Front. Immunol..

[36] Shu D.H., Ho W.J., Kagohara L.T., Girgis A., Shin S.M., Danilova L., Lee J.W., Sidiropoulos D.N., Mitchell S., Munjal K. (2024). Immunotherapy response induces divergent tertiary lymphoid structure morphologies in hepatocellular carcinoma. Nat. Immunol..

[37] Gunderson A.J., Rajamanickam V., Bui C., Bernard B., Pucilowska J., Ballesteros-Merino C., Schmidt M., McCarty K., Philips M., Piening B. (2021). Germinal center reactions in tertiary lymphoid structures associate with neoantigen burden, humoral immunity and long-term survivorship in pancreatic cancer. Oncoimmunology.

[38] Zhao L., Jin S., Wang S., Zhang Z., Wang X., Chen Z., Wang X., Huang S., Zhang D., Wu H. (2024). Tertiary lymphoid structures in diseases: Immune mechanisms and therapeutic advances. Signal Transduct. Target. Ther..

[39] Tang Z., Bai Y., Fang Q., Yuan Y., Zeng Q., Chen S., Xu T., Chen J., Tan L., Wang C. (2025). Spatial transcriptomics reveals tryptophan metabolism restricting maturation of intratumoral tertiary lymphoid structures. Cancer Cell.

[40] Fridman W.H., Meylan M., Pupier G., Calvez A., Hernandez I., Sautès-Fridman C. (2023). Tertiary lymphoid structures and B cells: An intratumoral immunity cycle. Immunity.

[41] Fridman W.H., Meylan M., Petitprez F., Sun C.M., Italiano A., Sautès-Fridman C. (2022). B cells and tertiary lymphoid structures as determinants of tumour immune contexture and clinical outcome. Nat. Rev. Clin. Oncol..

[42] Hauser M.A., Legler D.F. (2016). Common and biased signaling pathways of the chemokine receptor CCR7 elicited by its ligands CCL19 and CCL21 in leukocytes. J. Leukoc. Biol..

[43] Moser B. (2015). CXCR5, the Defining Marker for Follicular B Helper T (TFH) Cells. Front. Immunol..

[44] Sautès-Fridman C., Lawand M., Giraldo N.A., Kaplon H., Germain C., Fridman W.H., Dieu-Nosjean M.C. (2016). Tertiary Lymphoid Structures in Cancers: Prognostic Value, Regulation, and Manipulation for Therapeutic Intervention. Front. Immunol..

[45] Martinet L., Filleron T., Le Guellec S., Rochaix P., Garrido I., Girard J.P. (2013). High endothelial venule blood vessels for tumor-infiltrating lymphocytes are associated with lymphotoxin β-producing dendritic cells in human breast cancer. J. Immunol..

[46] Ager A. (2017). High Endothelial Venules and Other Blood Vessels: Critical Regulators of Lymphoid Organ Development and Function. Front. Immunol..

[47] Aoyama S., Noda T., Akita H., Mukai Y., Sasaki K., Hasegawa S., Yamada D., Tomimaru Y., Asaoka T., Takahashi H. (2025). Tumor-associated high endothelial venules are associated with enhanced lymphocyte infiltration and favorable prognosis in resected hepatocellular carcinoma. Cancer Immunol. Immunother..

[48] Li Y., Chen Y., Wu Z., Shi Y., Li M., Cai P., Zhang H., Liu C., Chen W., Li Q. (2026). Noninvasive MRI imaging feature-based prediction of intratumoral tertiary lymphoid structure maturity in hepatocellular carcinoma: A multicenter retrospective study. Eur. Radiol..

[49] Li H., Liu H., Fu H., Li J., Xu L., Wang G., Wu H. (2021). Peritumoral Tertiary Lymphoid Structures Correlate with Protective Immunity and Improved Prognosis in Patients with Hepatocellular Carcinoma. Front. Immunol..

[50] Zhang T., Lei X., Jia W., Li J., Nie Y., Mao Z., Wang Y., Tao K., Song W. (2023). Peritumor tertiary lymphoid structures are associated with infiltrating neutrophils and inferior prognosis in hepatocellular carcinoma. Cancer Med..

[51] Sarelin N., Kairaluoma V., Saarnio J., Kauppila J.H., Böhm J., Huhta H., Väyrynen J.P., Helminen O. (2026). Correction to: The prognostic value of peritumoural and intratumoural tertiary lymphoid structures in hepatocellular carcinoma. Virchows Arch..

[52] Gu Y., Guo Y., Gao N., Fang Y., Xu C., Hu G., Guo M., Ma Y., Zhang Y., Zhou J. (2022). The proteomic characterization of the peritumor microenvironment in human hepatocellular carcinoma. Oncogene.

[53] Koca D., Abedi-Ardekani B., LeMaoult J., Guyon L. (2024). Peritumoral tissue (PTT): Increasing need for naming convention. Br. J. Cancer.

[54] Li H., Wang J., Liu H., Lan T., Xu L., Wang G., Yuan K., Wu H. (2020). Existence of intratumoral tertiary lymphoid structures is associated with immune cells infiltration and predicts better prognosis in early-stage hepatocellular carcinoma. Aging (Albany NY).

[55] Hu L., Li X., Yang C., Zhou B., Du C., Jiang N. (2024). Prognostic value of tertiary lymphoid structures in hepatocellular carcinoma: A meta-analysis and systematic review. Front. Immunol..

[56] Zheng M., Tian Z. (2019). Liver-Mediated Adaptive Immune Tolerance. Front. Immunol..

[57] Ouyang F.Z., Wu R.Q., Wei Y., Liu R.X., Yang D., Xiao X., Zheng L., Li B., Lao X.M., Kuang D.M. (2016). Dendritic cell-elicited B-cell activation fosters immune privilege via IL-10 signals in hepatocellular carcinoma. Nat. Commun..

[58] Shu D.H., Ho W.J., Kagohara L.T., Girgis A., Shin S.M., Danilova L., Lee J.W., Sidiropoulos D.N., Mitchell S., Munjal K. (2023). Immune landscape of tertiary lymphoid structures in hepatocellular carcinoma (HCC) treated with neoadjuvant immune checkpoint blockade. bioRxiv.

[59] Li J., Zhang L., Xing H., Geng Y., Lv S., Luo X., He W., Fu Z., Li G., Hu B. (2024). The Absence of Intra-Tumoral Tertiary Lymphoid Structures is Associated with a Worse Prognosis and mTOR Signaling Activation in Hepatocellular Carcinoma with Liver Transplantation: A Multicenter Retrospective Study. Adv. Sci..

[60] Long S., Li M., Chen J., Zhong L., Dai G., Pan D., Liu W., Yi F., Ruan Y., Zou B. (2025). Transfer learning radiomic model predicts intratumoral tertiary lymphoid structures in hepatocellular carcinoma: A multicenter study. J. Immunother. Cancer.

[61] Xu J., Cheng Y., Feng Y., Wang Y. (2025). The Dichotomous Role of Tertiary Lymphoid Structures in Hepatocellular Carcinoma: From Spatial Location to Clinical Implications. Immunol. Investig..

[62] Long S., Li M., Chen J., Zhong L., Abudulimu A., Zhou L., Liu W., Pan D., Dai G., Fu K. (2024). Spatial patterns and MRI-based radiomic prediction of high peritumoral tertiary lymphoid structure density in hepatocellular carcinoma: A multicenter study. J. Immunother. Cancer.

[63] Liu L., Gao F., Li Y., Cheng J., Zhang H., Cai P., Chen W., Li X. (2025). An Interpretable Radiomics-Based Model Using Susceptibility-Weighted Imaging for Non-Invasive Prediction of Tertiary Lymphoid Structures in Hepatocellular Carcinoma. J. Hepatocell. Carcinoma.

[64] Sato Y., Silina K., van den Broek M., Hirahara K., Yanagita M. (2023). The roles of tertiary lymphoid structures in chronic diseases. Nat. Rev. Nephrol..

[65] Le Bihan D. (2019). What can we see with IVIM MRI?. Neuroimage.

[66] Surov A., Meyer H.J., Wienke A. (2017). Correlation between apparent diffusion coefficient (ADC) and cellularity is different in several tumors: A meta-analysis. Oncotarget.

[67] Chen W., DelProposto Z., Liu W., Kassir M., Wang Z., Zhao J., Xie B., Wen Y., Wang J., Hu J. (2014). Susceptibility-weighted imaging for the noncontrast evaluation of hepatocellular carcinoma: A prospective study with histopathologic correlation. PLoS ONE.

[68] Kurebayashi Y., Ojima H., Tsujikawa H., Kubota N., Maehara J., Abe Y., Kitago M., Shinoda M., Kitagawa Y., Sakamoto M. (2018). Landscape of immune microenvironment in hepatocellular carcinoma and its additional impact on histological and molecular classification. Hepatology.

[69] Rhee H., Cho E.S., Nahm J.H., Jang M., Chung Y.E., Baek S.E., Lee S., Kim M.J., Park M.S., Han D.H. (2021). Gadoxetic acid-enhanced MRI of macrotrabecular-massive hepatocellular carcinoma and its prognostic implications. J. Hepatol..

[70] Wu J., Zuo Z., Na L., Zhang W., Guo Y., Zhu Z., Ren Q., Peng W.K., Han L. (2025). Machine learning-driven prediction of intratumoral tertiary lymphoid structures in hepatocellular carcinoma using contrast-enhanced CT imaging and integrated clinical data. Front. Oncol..

[71] Wang R., Li Y., Zhang X., Yan L., Xu Z., Chen Z., Wu H., Jia W. (2026). Multi-phase CT-based intratumoral and peritumoral radiomics for predicting tertiary lymphoid structures of hepatocellular carcinoma: A multi-center retrospective cohort study. Eur. J. Surg. Oncol..

[72] Taouli B., Ba-Ssalamah A., Chapiro J., Chhatwal J., Fowler K., Kang T.W., Knobloch G., Koh D.-M., Kudo M., Lee J.M. (2024). Correction to: Consensus report from the 10th Global Forum for Liver Magnetic Resonance Imaging: Developments in HCC management. Eur. Radiol..

[73] Xie W., Sun L., Fu Y., Liu Y., Wang J., Liu L. (2026). Contrast-enhanced MRI for Hepatocellular Carcinoma Assessment: Advances in Contrast Agents and Immunotherapy. Acad. Radiol..

[74] Miar A., Arnaiz E., Bridges E., Beedie S., Cribbs A.P., Downes D.J., Beagrie R.A., Rehwinkel J., Harris A.L. (2020). Hypoxia Induces Transcriptional and Translational Downregulation of the Type I IFN Pathway in Multiple Cancer Cell Types. Cancer Res..

[75] Prior O., Macarro C., Navarro V., Monreal C., Ligero M., Garcia-Ruiz A., Serna G., Simonetti S., Braña I., Vieito M. (2024). Erratum for: Identification of Precise 3D CT Radiomics for Habitat Computation by Machine Learning in Cancer. Radiol. Artif. Intell..

[76] Li J., Qiu Z., Zhang C., Chen S., Wang M., Meng Q., Lu H., Wei L., Lv H., Zhong W. (2023). ITHscore: Comprehensive quantification of intra-tumor heterogeneity in NSCLC by multi-scale radiomic features. Eur. Radiol..

[77] Dong M., Jin X., Zhang L., Liang Y., Cao J., Li C., Huang M. (2026). Intratumoral Spatial Heterogeneity at Dynamic Contrast-Enhanced MRI for Assessing Tertiary Lymphoid Structures in Hepatocellular Carcinoma. J. Magn. Reson. Imaging.

[78] Li Y., Li X., Xiao X., Cheng J., Li Q., Liu C., Cai P., Chen W., Zhang H., Li X. (2025). A novel hybrid model for predicting tertiary lymphoid structures and targeted immunotherapy outcomes in hepatocellular carcinoma: A multicenter retrospective study. Eur. Radiol..

[79] Agoro R., Taleb M., Quesniaux V.F.J., Mura C. (2018). Cell iron status influences macrophage polarization. PLoS ONE.

[80] Huang C., Xiao X., Guo M., Hu X., Liu C., Wang J., Zhang H., Li X., Cai P. (2023). Value of susceptibility-weighted imaging in differentiating benign from malignant portal vein thrombosis. Quant. Imaging Med. Surg..

[81] Ma L., Liao S., Zhang X., Zhou F., Geng Z., Hu J., Zhang Y., Zhang C., Meng T., Wang S. (2025). Application of Intravoxel Incoherent Motion in the Prediction of Intra-Tumoral Tertiary Lymphoid Structures in Hepatocellular Carcinoma. J. Hepatocell. Carcinoma.

[82] Huang C.W., Lin S.E., Huang S.F., Yu M.C., Tang J.H., Tsai C.N., Hsu H.Y. (2022). The Vessels That Encapsulate Tumor Clusters (VETC) Pattern Is a Poor Prognosis Factor in Patients with Hepatocellular Carcinoma: An Analysis of Microvessel Density. Cancers.

